# Temporal and Spatial Melanoma Trends in Austria: An Ecological Study

**DOI:** 10.3390/ijerph110100734

**Published:** 2014-01-06

**Authors:** Daniela Haluza, Stana Simic, Hanns Moshammer

**Affiliations:** 1 Center for Public Health, Institute of Environmental Health, Medical University of Vienna, Kinderspitalgasse 15, Vienna A-1090, Austria; E-Mail: daniela.haluza@meduniwien.ac.at; 2 Institute for Meteorology, University of Natural Resources and Life Sciences, Peter-Jordan-Strasse 82, Vienna A-1190, Austria; E-Mail: stana.simic@boku.ac.at

**Keywords:** malignant melanoma, UV radiation, Alpine region, metrology, stratospheric ozone

## Abstract

Annual solar ultraviolet radiation (UVR) is mostly determined by latitude and altitude. Over the last decades, increasing UVR ground levels have been observed. Exposure to UVR is associated with a life-time risk to develop melanoma, a malign skin cancer. Thus, we hypothesized that melanoma incidence in Austria is associated with altitude of place of living and time of diagnosis. We investigated this hypothesis in an ecological study by district and year for Austrian melanoma incidence (1990–2010) and mortality (1970–2011) data. As expected, incidence rates increased with altitude (about 2% per 10 m) and year (about 2%). Additionally, melanoma incidence rates were about 50% higher in urban than in rural districts. In contrast, mortality rates decreased with altitude (for males: 0.4% per 10 m, for women: 0.7% per 10 m, respectively). The observed discrepancy between incidence and mortality data could partly be explained by melanoma diagnosis at earlier tumor stage in districts with higher altitude. Possible reasons for this finding include higher awareness of patients, better diagnostic performance of medical professionals working at higher altitudes, or slower tumor growth due to protective effects of sun light-associated vitamin D synthesis.

## 1. Instruction

In general, risk factors for the development of melanoma skin cancer comprise genetic factors, with skin type being the most visible and obvious one, as well as behavioral factors and natural and artificial ultraviolet radiation (UVR) [1,2,3,4,5,6,7]. Solar light is the most important UVR source [8]. Recreational sun exposure is a melanoma risk preventable by life-style modifications addressed by public (skin) health-promoting efforts [9,10,11]. In addition to avoidance of direct sunlight, other recommended sun protective measures include the use of broad-spectrum sunscreens and wearing protective clothes, sunglasses, and hats [12]. Thus, time spent outdoors around and after noon on sunny days and resulting sunburns are key drivers of sun exposure effects attributable to behavior [13]. So far, information is lacking whether sunburns are a proxy for less health-protective behavior, cumulative UVR exposure or inflammatory reactions that pose a melanoma risk [11]. Blistering sunburns in childhood or adolescence are highly predictive for melanoma onset later in life, as shown in a comprehensive literature review conducted by Oliveria *et al.* [14]. Nevertheless, early and late onset melanoma could have different causes. While younger age melanomas were shown to be influenced by gender-specific hormone status and gestational factors such as birth weight, older-age cancers could be rather due to cumulative life-time UV-light exposure [15,16].

Besides temporal variation in solar activity, ground level UVR depends on stratospheric ozone layer thickness, geographic characteristics e.g., latitude and altitude, and weather patterns [17,18,19,20]. Changes regarding both cloud cover and stratospheric ozone strongly impact the long-term ground level UVR and are subject to the anthropogenic climate change. Variability in cloudiness most obviously affects local ground exposure, whereas larger-scale meteorological phenomena influence regional ozone layer thickness, and thus have an impact on erythemal UVR. 

Recently, increased intensity and frequency of so-called ozone mini-holes have been observed all over the European continent [21,22,23]. Characteristically, these ozone mini-holes have the size of several hundred kilometers (e.g., covering a substantial part of the Alps in Middle Europe) and persist for several days, mostly in springtime. As skiing at higher altitudes is quite popular at this time of the year, effective solar radiation protective measures are important for outdoor athletes [24,25]. 

Like other countries, in Austria, melanoma incidence rates have increased over time (1992: 8.1; 2009: 10.6 per 100,000 standardized to WHO 2001 world population, respectively). Nevertheless, melanoma mortality rates (around 2–2.4/100,000) remained fairly constant [26]. 

So far, retrospective investigations of spatial variations of Austrian melanoma data are missing. Thus, the current ecological study aimed at exploring temporal and spatial trends of melanoma incidence and mortality rates in respect to altitude of place of residence. In Austria, routine health data are usually available per district and UVR dose data only for nine locations. In the current study, we assumed a latency period for melanoma of at least 10 years based on the respective literature, with altitude and inter-annual variability as main predictors of melanoma risk-associated UVR exposure [27,28,29,30,31].

This analysis of skin cancer trends was part of the interdisciplinary project “UVSkinRisk—Health at risk through UVR-induced Skin Cancer in the Context of a Changing Climate” supported by the Austrian Climate Research Programme (ACRP). 

The UVR monitoring and modeling part will be elaborated on in a consecutive report. In short, UVR model data indicated an increase in UVR (erythemal weighted daily dose in kJ/m²) over time with the strongest increase in the period 1977–1996. In the years before 1970, the annual UVR amounted to about 500 kJ/m² (erythemal dose) in or near the capital city, Vienna (160 m above sea level). In the period 1960–2010, the annual average increase of this dose was about 1.2 kJ/m² per 10 m, the annual erythemal dose increased by about 1.3 kJ/m². The slope of the increase from 1977 to 1996 slightly differed between Austrian monitoring stations with the highest relative changes at eastern and southeastern stations. Although these observations of differences between slopes could mostly be explained by differences in cloudiness patterns, stratospheric ozone was the dominant factor responsible for long-term general UVR increases. The small differences between monitoring stations were not captured well by the model data and thus could not be incorporated in the epidemiological study.

## 2. Experimental Section

### 2.1. Melanoma Data

The International Classification of Diseases (ICD) is the standard diagnostic tool to code and classify mortality data. During the decades, coding of “cause of death” changed. ICD-8 (ICD 8th revision) was implemented in 1979. Regarding skin cancers, ICD-8 was equivalent to ICD-9, which was employed from 1980 to 2001 (172: Malignant melanoma of skin; 173: Other malignant neoplasm of skin). However, since 2002, ICD-10 has been used (C43: Malignant melanoma of skin; C44: Other malignant neoplasm of skin). The 4-digit code also provides information on primary tumor location. However, it codes as “9” indicating “not specified” in more than 90% of all cases. Individual mortality data by age in years, sex, cause of death, date of death, and home district were available from 1970 to 2011. As cancer incidence data and census data are only freely available in 5-year age groups, we also assigned mortality data to 5-year age groups.

For the years 1990 to 2010, the Austrian cancer register provides incidence data for melanoma, but not for non-melanoma skin cancers. Available data include age in 5-year age-groups, sex, diagnosis (ICD-10), stage at diagnosis, year of diagnosis, and home district. More detailed data are not available because of confidentiality concerns. 

While the mortality register is practically complete, the cancer register depends on the obligatory, but not fully obeyed reporting of medical professionals. Only cancers that lead to death in a high percentage of cases are included in that registry as sufficient reporting can be controlled for by verifying data from the mortality register with the cancer register. Good quality of the reporting system is assumed if at least 90% of all cancers found through the mortality register have already been registered in the cancer register. Currently, the Austrian cancer registry is supposed to be 94.2% complete for all included types of cancer overall [26]. However, according to Richtig and co-workers hospitals located in the Austrian federal country of Styria did not fully report melanoma cases for several years [32,33]. 

In general, all cancer cases should be reported in the year of initial diagnosis. Delays in reporting are caused by several factors such as histological confirmation of clinically manifest cancers. Some additional malignancies never get reported and are only discovered in the mortality register. Regarding analysis of trends, it should be noted that cancer registers of recent years are usually not yet as complete as data from earlier years. 

### 2.2. Age-adjustment

Austrian census data were collected every 10 years (1971, 1981, 1991, and 2001). Population numbers were extracted per district, sex and 5-year age group. For the remaining years, numbers were estimated by linear interpolation. Annual cases per district were standardized according to the age and sex distribution of the total Austrian population in the year 1991.

### 2.3. Independent Factors

The following four independent factors were explored: (1) Height above sea level in meters of the home district by altitude of the respective district capital. Especially with mountainous districts where the capital is already situated at higher altitude, habitable areas of the district often reach much higher altitudes. Still, the majority of inhabitants are supposed to live in or near the district capital. (2) Year of diagnosis (for incidence) and death (for mortality data), respectively. (3) “Urban living”: Austria (approx. 8 million inhabitants) consists of 121 districts. Twenty-three of these districts are assigned to the city of Vienna (1.8 million inhabitants) and thus, classified as urban areas. Fourteen larger Austrian cities with approximately 20,000 to 300,000 inhabitants are defined as single districts each, and the surrounding areas form different districts. Many smaller towns are district capitals that also include the surrounding smaller towns, villages, and countryside. Single town districts and the city districts of Vienna were each indicated by a dummy variable discerning 84 “rural districts”, 14 “urban districts” and 23 “districts of Vienna”. (4) “Styrian factor” (for incidence data only): Because of the clear evidence of under-reporting in Styria, a special dummy was introduced for the 17 Styrian districts [32,33]. Based on inspection of the raw data and communication with Statistics Austria, this dummy flag was set for Styrian districts before the year 2005.

### 2.4. Regression Models

Poisson regression models were applied to investigate how the independent factors affected melanoma incidence and mortality. In a first step, regarding melanoma incidence, we analyzed separate Poisson models for each year with age-adjusted case numbers (dependent variable) and altitude (independent variable), offset by district population. This was done in the pilot phase of the study when only incidence data up to the year 2004 were available. Second, concerning melanoma incidence rates for each sex (male and female) and 5-year age groups, we evaluated separate Poisson regression models with the outcome case numbers per district and year (raw numbers), and year, altitude, urban factors, and Styrian factor (independent variables), offset by population number in the specific age group of each district. For melanoma deaths, separate Poisson models for each sex were evaluated using the age-adjusted case numbers as dependent variable and altitude, year and urban factors as independent variables, offset by district population. Further, we investigated deviations from linearity in General Additive Models (GAM). In addition, we examined distribution of melanoma stages at diagnosis by sea level using sensitivity analyses. After performing arcsine transformation on the proportion of each stage, we investigated the effect of altitude and urbanity of the home district on the transformed proportion controlling for the (transformed) proportion of missing data by means of linear regression analyses. All analyses were performed using STATA Statistical Software, version 12 (StataCorp, College Station, TX, USA). For all statistical analyses, level of significance was set at 5%.

## 3. Results

**Figure 1:**
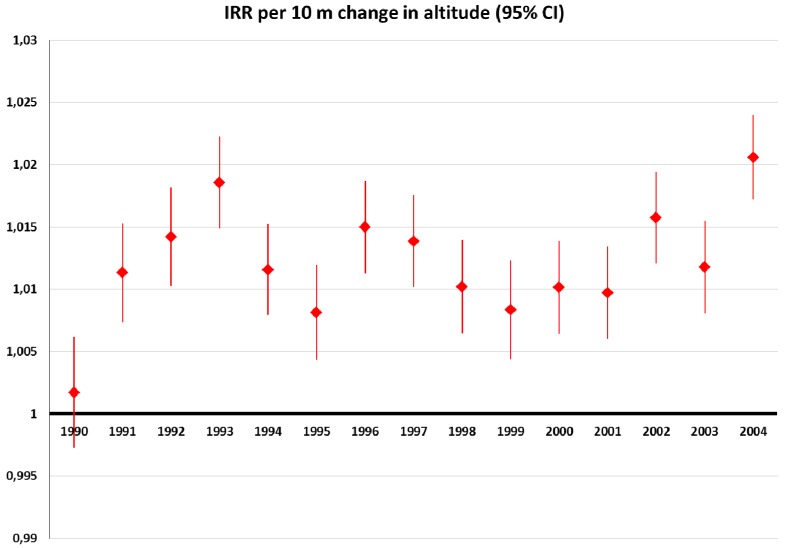
Annual melanoma incidence risk ratios (IRR) per 10 m increase in altitude: Age-adjusted data, controlled for urban *versus* rural districts, and offset by district population (1991 census).

In all years, except 1990, age-adjusted melanoma incidence significantly increased with altitude with point estimates ranging between 0.5 and 1% increase in risk per 10 m (data not shown). In 1990, we observed a (non-significant) decrease in melanoma risk with altitude. In this analysis, we consolidated urban districts with districts from Vienna. Urban districts displayed a markedly higher incidence than rural districts. Since urban agglomerations tend to be at lower altitudes, inclusion of the urban factor slightly strengthened the adverse effect of altitude. Thus, we observed a (non-significant) increase in incidence with altitude also in the year 1990 (Figure 1).

**Figure 2:**
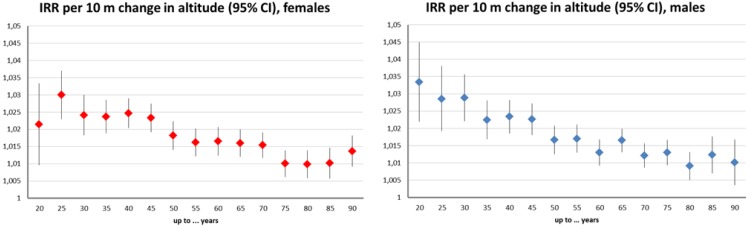
Age-group specific melanoma incidence risk ratios (IRR) and 95% confidence intervals (CI) per 10 m increase in altitude: Raw incidence data controlled for year, urban and “Styrian” factors, and offset by the same year population number of the very group; females (left), males (right).

**Figure 3:**
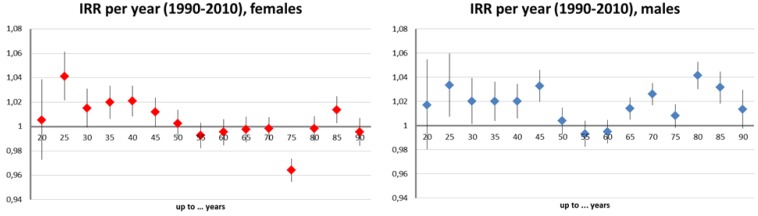
Age-group specific melanoma incidence risk ratios (IRR) and 95% confidence intervals (CI) per year (1990-2010): Raw incidence data controlled for altitude, urban and “Styrian” factors, and offset by the same year population number of the very group; females (left), males (right).

The models investigating melanoma incidence per age group found an increased risk with altitude for all age groups (Figure 2). This effect was more pronounced in the younger age groups before the age of 45 and slightly stronger in men (approx. 3% increase in incidence per 10 m in the “below 30” age-group) than in women (approx. 2.5%). All effect estimates were significant at the 5% level. Most age groups of both genders displayed an increase in incidence over time (Figure 3). Additionally, this trend was positively significant (around 2% per year) in the aforementioned younger age groups with the exception of the youngest. This temporal trend was clearly more pronounced among males.

**Figure 4:**
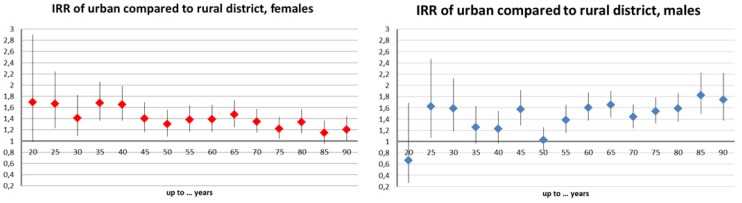
Age-group specific melanoma incidence risk ratios (IRR) and 95% confidence intervals (CI) in rural districts compared to rural districts: Raw incidence data controlled for altitude, year, and “Styrian” factors, and offset by the same year population number of the very group; females (left), males (right).

**Figure 5:**
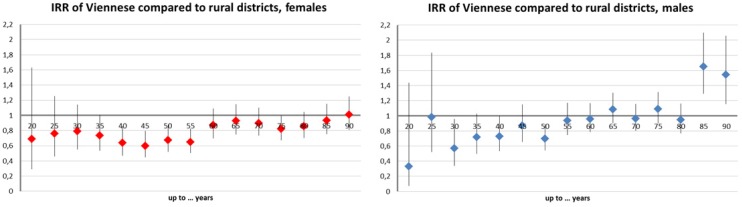
Age-group specific melanoma incidence risk ratios (IRR) and 95% confidence intervals (CI) in Viennese districts compared to rural districts: Raw incidence data controlled for altitude, year, and “Styrian” factors, and offset by the same year population number of the specific group; females (left), males (right).

Melanoma risk was higher for urban districts compared to rural districts (Figure 4). Estimates were significant in the majority of age groups and ranged between 20% and 80%. The districts of Vienna displayed a smaller risk than rural districts (Figure 5). With a 50% reduction between 30 and 55 years, this effect was most pronounced in middle age groups. Further, the Styrian factor proved spuriously protective as expected (−80% to −40%, data not shown).

Age-standardized melanoma mortality rate ratios (MRR) increased over time, although not very much and a leveling off and even decrease (regarding women) was evident at the end of the observation period in GAM using cubic smoothing splines. Urban districts showed higher mortality rates than rural districts. Contrary to incidence data, melanoma deaths were more likely in Vienna and declined with increasing altitude (Table 1).

After performing arcsine transformation of the proportion of each tumor stage, we investigated the effect of altitude and urbanity of the home district on the transformed proportion controlling for the (transformed) proportion of missing data by means of linear regression. The coefficient for the proportion of missing values was always significant and negative. Further, percentage of stage 2 cases at diagnosis was significantly higher with increasing altitude while percentage of stage 4 and DCO cases was significantly smaller.

**Table 1:** Age-standardized melanoma mortality rate ratios (MRR) and 95% confidence intervals (CI) for selected risk factors according to logistic regression (for the years 1970–2011).

	Men		Women	
Factor	MRR	95% CI	MRR	95% CI
Per year	1.010	1.008–1.013	1.013	1.011–1.016
Per meter	0.9996	0.9994–0.9998	0.9993	0.9991–0.9995
Urban *vs*. Rural	1.167	1.074–1.268	1.195	1.100–1.300
Vienna *vs*. Rural	1.206	1.109–1.313	1.247	1.149–1.354

## 4. Discussion

### 4.1. The Discrepancy between Incidence and Mortality

In Austria, a strong increase in UVR dose was seen between 1977 and 1996. Assuming a melanoma onset latency of at least 10 years, for studying national temporal melanoma trends, this period is in good accordance with availability of melanoma incidence data (1990–2010). Also, 21 years seemed adequately long to investigate respective spatial differences by altitude. 

Originally, our study hypothesis only concerned melanoma incidence data. Regarding mortality, we already observed a slowing down or even reversal of the temporally increasing trend in the raw data. According to Lee and co-workers, reduced melanoma deaths could rather be caused by recent improvements in diagnosis and therapy than reversing trends in causal factors [34]. Therefore, mortality data could be less suitable to study temporal trends. But, being surprised by the exceptional strength of association between estimated change in UV dose and increase in incidence rates, we also analyzed mortality data in a second step.

Regarding incidence data patial and temporal were highly compatible with the respective changes in UVR. A *priori*, we assumed that melanoma cases at younger ages would display less exposure misclassification. Because latency should necessarily be shorter in early onset cancers, home district at time of diagnosis should be more closely related to the district at time of cancer initiation [29,35,36,37,38]. A similar argument holds for the temporal trend where a shorter latency period of about 10 years would lead to a closer coincidence between high UVR increase and supposed initiation period [27]. Only for cases occurring in very young patients, we expected wider confidence intervals due to fewer cases and lower point estimates caused by competing genetic and/or embryonic influences [5,6]. Indeed, effect estimates per 10 m and per year were fairly similar for the younger age groups up to below 45 or 50 years. Both measures also reflected comparable increases in annual UVR. 

Nevertheless, it seemed implausible that a 0.2% increase in solar radiation should lead to an approximately 2% increase in melanoma incidence rates. We assumed that a non-differential error in the estimation of UVR exposure by altitude could not fully explain such a large spatial effect: In mountain districts, the central town often is situated on the bottom of a valley, while the total habitable area reaches to higher altitudes. Additionally, the citizens of this central town could spend more leisure time on outdoor activities in higher mountain regions, simply due to easy accessibility. The temporal association between increasing UVR and melanoma incidence could still be confounded by other temporal trends, especially behavior changes. The importance of behavioral factors for temporal trends is also supported by the differences in time trends between men and women.

**Figure 6:**
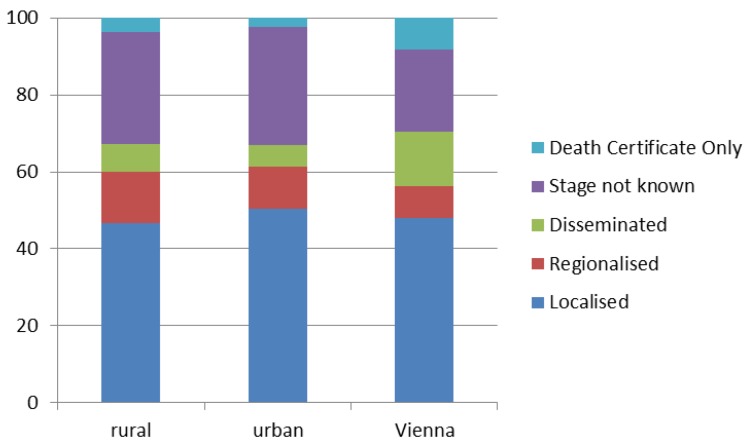
Distribution of melanoma cancer stages at time of diagnosis (in %): Comparison of different types of districts (rural, urban, and Vienna).

The discrepancy between the two outcomes (melanoma mortality and incidence rates) regarding the influence of altitude as well as observations regarding Viennese data was unexpected. Subsequently, we analyzed “stage at diagnosis” in order to get more insight into these striking findings. We compared rural, urban, and Viennese districts. According to Figure 6, more cancer cases in Vienna were diagnosed only after the death of the patient (Death Certificate Only, DCO), and also in the dissemination stage (stage 4). This finding could partly explain the reported lower melanoma incidence in Vienna. What is more, apart from behavioral differences, also a higher proportion of people with migratory background and thus darker skin types could be responsible for lower melanoma incidence rates in the capital Vienna. Nevertheless, these associations could not explain higher melanoma mortality rates.

Further, we assessed the influence of altitude on melanoma stages at time of diagnosis, revealing an earlier diagnosis in districts at higher altitude with significantly higher percentage of melanoma cases diagnosed in stage 2 and significantly lower cases in stage 4 and as DCO. We also found more cases at stage 4 and DCO in Vienna, although this difference was not significant in the multiple regression models. Concerning temporal trends, “year of diagnosis” was no significant predictor of any stage after controlling for “missing values”, while the percentage of “missing values” increased over time (data not shown).

Access to health care might differ between some sub-groups living in Vienna (e.g., migrants) and the remaining Austrian population. On the other hand, the impact of altitude on stage at diagnosis seems to be less obvious. However, as a possible explanation, awareness of local medical professionals of various disciplines due to higher melanoma risk could trigger better diagnostic coverage at higher altitude. Tumor thickness defined by the Breslow’s depth of invasion was shown to be the most critical survival predictor for melanoma survival in the Austrian population [39]. Therefore, earlier melanoma diagnosis in districts at higher altitudes could partly explain the lower mortality rate. 

As alternative explanation, higher exposure to UVR could increase endogenous vitamin D production [40]. There is some evidence that UV light exposure might protect against malignancies in general and melanoma specifically, indicating a divergent effect of UVR on skin cancer initiation and promotion [41,42,43]. However, as the respective literature is still contradictive and inconclusive, the association between vitamin D and cancer prevention, mortality reduction, and associated longevity needs further investigation [44,45,46,47]. Due to the well-known carcinogenic properties of solar light, unprotected intentional UV light exposure should not be used for enhancing vitamin D levels, as Gilaberte and co-workers (2011) concluded in their recently published review [48].

Earlier melanoma diagnosis could also lead to a spurious increase of incidence in younger age groups or age-adjusted analysis, because melanoma incidence increases strongly with age. Theoretically, this could even lead to an artificial impact of district altitude on incidence risk. Thus, for sensitivity analysis, we performed a logistic regression on melanoma incidence using not age-adjusted raw data (per year, district, and sex). The results of this regression confirmed that cancer incidence increased over time and was higher in districts at higher altitude (data not shown). Relative incidence was also higher in urban compared to rural districts. In Vienna, the incidence was lower than in (other) urban districts, but higher than in rural districts (significantly for males). 

This crude analysis could be biased towards higher incidence with higher altitude if also life expectancy increases with altitude. However, this was not the case with Austrian districts. On the contrary, a simple analysis of age at death per altitude of district revealed that in the years 1990–2011, this parameter decreased significantly but weakly with altitude (−0.02 years per 10 m). Age at death was on average nearly one year higher both in urban districts (0.97) and in Vienna (1.0). So, although in general, raw data (not age-adjusted) could be subject to bias, this analysis clearly demonstrated that the increase in incidence with altitude was not an artifact caused by earlier detection.

### 4.2. Implications for Climate Change-Related Health Impact Assessment

Health impact assessments are usually driven by mortality risks because, at least with approaches based on “willingness to pay”, deaths by far outweigh any other costs of disease [49]. Since the reasons for the reduced risk of melanoma deaths at higher altitudes are unknown, unambiguous conclusions could not be drawn. 

In the present study, we observed an increasing melanoma incidence trend over time. This increasing trend seems too strong to be entirely explained by the increase in annual cumulative UVR. Additionally, the increase in day-to-day variability, although only poorly captured by the UVR reconstruction model, might well play an even more important role. Furthermore, behavioral changes could probably be the main reasons for this increasing trend which is most clearly documented by the much higher risk in urban districts compared to rural ones. However, urbanity could be considered as a poor proxy for skin health risky behavior although compared to the rural population the urban population might experience more intermittent UV light exposure as a risk factor for melanoma [50]. Furthermore, our definition of rural and urban districts could also be subject to measurement errors. So, the precise impact of behavior could be much more important than indicated by the modeled urban effect. 

Many factors could affect future sun protective behavior. To date, it is not entirely clear how and to which extent climate change will affect individual UVR exposure habits. As an example, higher temperatures could make people wear less protective, lighter clothing or seek shade more frequently. Thus, assessing the impact of climate change on melanoma risk as a multi-causal, complex mechanism should be investigated in elaborated study settings worldwide.

UVR trends are difficult to predict as local exposure strongly depends on cloud cover and meteorological modeling approaches. Even regional meteorological phenomena that affect so-called ozone mini-holes are only poorly depicted in current models. According to den Outer and colleagues (2010), cloud cover contributed to as much as two-thirds of changes in total UVR measured in European regions [19]. Nevertheless, according to a more recent paper of the same working group employing the climate model (E39C-A), natural UVR was mostly driven by ozone layer changes, suggesting to continue monitoring and modeling efforts [20].

## 5. Conclusions

So far, the present study is the first approach assessing both melanoma incidence and mortality in the same population using an ecological approach. Melanoma incidence rates increased over time in Austria. Causative factors could include natural UV light, an increase of its day-to-day variability, and behavioral changes of individuals. All these phenomena are partly linked to climate change.

Living in urban areas compared to rural areas in Austria is associated with a substantially higher melanoma risk that could be caused by intermittent UVR exposure patterns.

Further, we found increased melanoma incidence, but not mortality rates for Austrian inhabitants living at higher altitudes. A possible explanation of this discrepancy could be that UVR induces melanoma, but also slows down tumor progression by means of elevated serum vitamin D levels. Although Shipman *et al*. also reported lower melanoma mortality in sunnier European countries, we suggest that this question should be addressed in further investigations [42]. 
